# Case report: Artemis deficiency and 3M syndrome—coexistence of two distinct genetic disorders

**DOI:** 10.3389/fped.2023.1211254

**Published:** 2023-07-13

**Authors:** Ayca Ceylan, Ilyas Emre Tekdemir, Nadir Kocak, Ivan Kingyue Chinn, Jordan Scott Orange, Hasibe Artac

**Affiliations:** ^1^ Department of Pediatrics, Division of Immunology and Allergy, Faculty of Medicine, Selcuk University, Konya, Turkey; ^2^ Department of Medical Genetics, Faculty of Medicine, Selcuk University, Konya, Turkey; ^3^ Department of Pediatrics, Division of Immunology, Allergy and Retrovirology, Baylor College of Medicine, Houston, TX, United States; ^4^ Department of Pediatrics, Presbyterian Morgan Stanley Children's Hospital, Columbia University, NY, United States

**Keywords:** ARTEMIS deficiency, 3M syndrome, immunodeficiency, whole-exome sequencing, coexistence of genetic disorders

## Abstract

The presence of two different genetic conditions in the same individual is possible, especially in populations with consanguinity. In this case report, we present the coexistence of Artemis deficiency (OMIM 602450) and Three M (3M) syndrome (OMIM 273750). A 10-months-old male patient with neuromotor developmental delay was evaluated for immunodeficiency due to recurrent respiratory infections diarrhea and oral moniliasis from the age of 1.5 months. He had facial dysmorphism with rotated ears, flat nose and hypertelorism. Neurological examination revealed generalized hypotonia and mental motor delay. Immunological screening of the patient demonstrated mild lymphopenia, hypogammaglobulinemia, reduced number of CD3^+^ T cells (980 cells/mm^3^) and CD19^+^ B cells (35 cells/mm^3^). He was diagnosed with leaky T^−^B^−^NK^+^ SCID. Exome sequence analysis showed the presence of a homozygous pathogenic *DCLRE1C* variant [c.194C > T; p.T65I (NM_001033855)] and a homozygous pathogenic variant in *OBSL1*, a gene associated with 3M syndrome [c.3922C > T; p.R1308X (NM_001173431)]. Our proband died of sepsis and multiple organ failure. This case illustrates that different clinical findings in patients might not be explained with a single genetic defect, and consanguinity increases the change for coexistence of autosomal recessive diseases. Clinicians should consider exome sequencing to identify disease-causing mutations in patients with heterogeneity of clinical findings.

## Introduction

Severe combined immunodeficiency (SCID) is a rare disorder characterized by profoundly defective T lymphocyte differentiation with or without abnormal development of B or NK lymphocytes or more rarely of the myeloid lineage and, presenting in infancy with life-threatening bacterial, fungal and viral infections infections (1, 2). SCID can be subdivided into T^+^B^+^, T^−^B^+^ or T^−^B^−^ SCID according to the different genetic forms affecting T or B lymphocytes. Artemis deficiency is the most common form of radiosensitive SCID (3). Pathogenic variants in *DCLRE1C*, encoding Artemis, cause a block in T-and B-cell development and confer sensitivity to ionizing radiation (4). Artemis codes DNA Double-Strand Break Repair/Variable (V), Diversity (D), Joining (J) Recombination Protein of T-cell receptor genes and immunoglobulin in T and B cell development (5). Therefore loss of Artemis gene activity leads to impaired recombination and causes T^−^B^−^ SCID. Also, hypomorphic mutations in Artemis can cause atypical SCID, hyper IgM syndrome, Omenn syndrome and inflammatory bowel disease (3).

Three M (3M) syndrome [Online Mendelian Inheritance in Man (OMIM) 273750] is a rare autosomal recessive disorder and characterized by severe pre- and postnatal growth deficiency, facial dysmorphism, large head circumference and normal intelligence (6). 3M syndrome is caused by mutations in three genes (Obscurin-like 1 [OBSL1]; Cullin 7 [CUL7]; Protein 8-containing helix-coiled domain [CCDC8]). These genes interact with each other to form the 3M complex that maintains microtubule and genome integrity (7). Deletion or destruction of any 3M gene and disruption of the 3M complex cause severe microtubule damage, abnormal chromosome segregation and cell death (7). The treatment for 3M syndrome is supportive and based on the patient’s symptoms.

Although rare, the presence of two different genetic conditions in the same individual is possible, especially in populations with consanguinity. Exome or whole genome sequencing is critical for correct diagnosis and optimal management of these diseases in cases whose clinical findings cannot be explained by a single cause. In this case report, we present the coexistence of Artemis deficiency (OMIM 602450) and Three M (3M) syndrome (OMIM 273750).

## Case description

**Figure 1:**
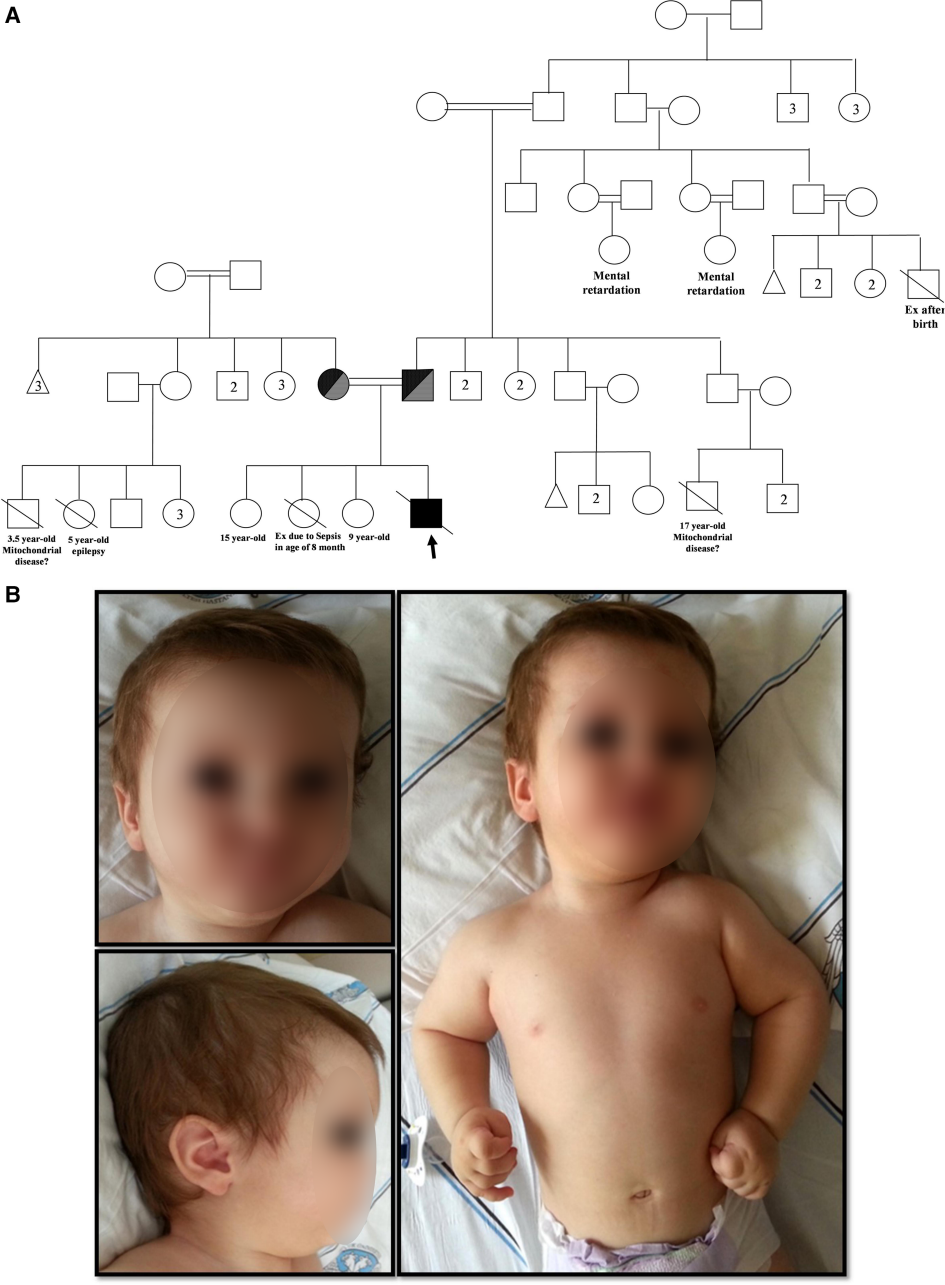
(**A**) Family pedigree chart. 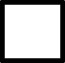 Male, 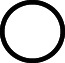 Female, 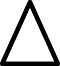 Abortus, 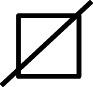 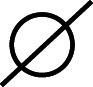 Deceased, 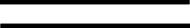 Consanguineous marriage, 
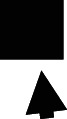
 Homozygous *DCLRE1C + OBSL1* (Proband), 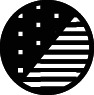 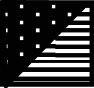 Compound heterozygous *DCLRE1C + OBSL1*. Numbers inside symbols indicate number of individuals. (**B**) Physical appearance of the patient (posteriorly rotated ears, low nasal bridge, flat nose, retromicrognathia, hypertelorism).

A 10-month-old male with neuromotor developmental delay was evaluated for immunodeficiency due to recurrent respiratory infections, diarrhea, and oral moniliasis from the age of 1.5 months. He was delivered at 34 weeks with a birth weight of 2,300 g, and he had no clinical history of hypoxia while in the neonatal intensive care unit. Neurological evaluation due to less interaction with surroundings and unable to sitting by 4 months was done. Hypotonia and exaggerated deep tendon reflexes on lower limbs were observed. His cranial MR imaging showed that non-specific hyperintensity in white matter. Metabolic screening (homosistein, amino acids in urine and blood samples, tandem mass spectrometry) was normal. He was the fourth child of consanguineous parents (Figure 1A). His sister died at the age of 8.5 months due to sepsis.

On physical examination, he had facial dysmorphism with posteriorly rotated ears, flat nose, and hypertelorism (Figure 1B). Neurological examination revealed generalized hypotonia and developmental motor delay. In immunological testing, mild lymphopenia, hypogammaglobulinemia, and reduced numbers of CD3^+^ T cells (980 cells/mm^3^) and CD19^+^ B cells (35 cells/mm^3^) were detected (Table 1). T lymphocyte activation with phytohemagglutinin was half of the control. Memory phenotype CD4^+^CD45RO^+^ T cells represented 58% of circulating CD4^+^ T cells, but no evidence of maternal engraftment was observed upon HLA-typing of this patient and his mother. The diagnosis was felt to be consistent with leaky T^−^B^−^NK^+^ severe combined immunodeficiency (SCID) (8). He was placed on trimethoprim-sulfomethoxazole (5 mg/kg/d, PO, daily) and fluconazole (6 mg/kg/d, PO, daily) for prophylaxis and on intravenous immunoglobulin replacement (0.5 g/kg/dose every 3–4 weeks). Still, the proband died from pneumonia, sepsis, and multiple organ failure at 21 months of age. Informed consent was obtained from the patient’s parents for publication.

**Table 1:** Immunological characteristics of the patient.

	10 months old	18 months old	Normal Value
Absolute lymphocyte count (/mm^3^)	2,650	2,190	3,200–10,800
Lymphocyte subsets (% and absolute values) (/µl)			
CD3^+^ T cell	37.1 (%)	33.6	51–79
	980	905	2,400–8,100
CD3^+^CD4^+^ T cell	13.6 (%)	13.4	31–54
	360	361	1,400–5,200
CD3^+^CD8^+^ T cell	16.1 (%)	13.4	10–31
	427	361	600–3,000
CD19^+^ B cell	1.4 (%)	2.8	14–44
	35	75	500–3,600
CD16^+^56^+^ NK cell	33.2 (%)	-	5–23
HLA-DR^+^	42.4 (%)	-	15–48
TCR-α*β*/CD3^+^	85.7	-	
TCR-*γδ*/CD3^+^	13	-	
CD45RA/CD3^+^	18.9	-	
CD45RO/CD3^+^	69.8	-	
Serum Immunoglobulin levels			
IgG (mg/dl)	357	767 (with IVIG)	499.2–628.6
IgM (mg/dl)	22.8	35.3	67.7–94.4
IgA (mg/dl)	5.97	6.67	21–37.7
IgE (IU/ml)	18.1	5	<50
Anti-tetanus toxoid antibody levels (IU/ml)	0.40	-	>0.15 IU/ml
Anti-HBS (mIU/ml)	4.17	-	>10

Exome sequencing of a premortem blood sample identified the presence of a homozygous *DCLRE1C* variant [c.194C > T; p.T65I (NM_001033855)] and a novel homozygous pathogenic variant in *OBSL1*, a gene associated with 3M syndrome [c.3922C > T; p.R1308X (NM_001173431)] (Supplementary Figure). The hypomorphic p.T65I mutation is known to result in reduced Artemis expression and either atypical or leaky SCID (9).

## Discussion

Affected residual Artemis expression and/or function lead to a broad spectrum of phenotypes at the clinical, cellular and immunological level. The expression level and function of residual protein caused by a hypomorphic mutant is important in determining the prognosis and severity of immunodeficiency (10).

Classical SCID patients cannot live beyond infancy without hematopoietic stem cell transplantation (HSCT), but some children with Artemis deficiency can manage to recover from infections in the first years of life, and symptoms may delay until adulthood in some individuals with hypomorphic Artemis mutations (3). However, progressive degradation of immunity and increased organ damage cause impairment of survival. As a new gene therapy for Artemis-deficient SCID, Cowan et al. proposed the novel lentiviral construct AProArt, containing human *DCLRE1C* cDNA driven by the *DCLRE1C* promoter sequence in their study (11).

In the study of Ghadimi et al. in nine patients with *DCLRE1C* mutation, reported that there was consanguinity in 7 patients, and the most typical first presentation were pneumonia, otitis media, BCG lymphadenitis and gastroenteritis, respectively (12). Also, 33.3% of the patients had a family history of spontaneous abortion. Our case also had a family history of spontaneous abortion. The study conducted by Lee et al. including 2 patients with combined immunodeficiency (CID) and 12 patients with Artemis-deficient CID from previous other studies, reported that patients having hypomorphic mutations with residual Artemis expression, V(D)J recombination or double-stranded DNA repair capacity had significant morbidities such as autoimmunity, recurrent infections, EBV-related lymphoma and carcinoma (3). In their study, 9 patients underwent HSCT, 6 patients survived, and 4 patients who did not receive HSCT died. Early HSCT should be considered to prevent poor survival in Artemis deficiency. Inoue et al. reported in their study that 8 patients with Artemis-SCID had missense variants in 2 patients, large genomic deletions in 5 patients, and one patient with compound heterozygous for one missense variant and large genomic deletion (13). Eight patients underwent allogeneic HSCT and two patients died of complications after HSCT.

Physical examination features of 3M syndrome include short broad neck and thorax, deformed sternum, square shoulders, prominent trapezii, hyperlordosis, prominent heels, short fifth fingers, loose joints, and skeletal changes including tall vertebral bodies and long slender tubular bones (6). This patient had dysmorphic features including frontal bossing, bulbous nose, broad forehead and triangular face. OSBL1 homozygous mutation lead to stop codon was detected. So, this additional mutation made confused to the clinician for accurate diagnosis before genetic analysis.

We detected a homozygous *DCLRE1C* variant (c.194C > T; p.T65I) and a homozygous pathogenic *OBSL1* variant in (c.3922C > T; p.R1308X). Volk et al. reported in three index patients with homozygous variants in *DCLRE1C* and nine patients from the same geographic region with homozygous or compound heterozygous *DCLRE1C* mutations in their study (9). There is no previously reported case with c.3922C > T; p.R1308X pathogenic variant.

Reports of multilocus variation causing blended phenotypes which are in 5% of the genetic diagnoses remain very limited (14). Chinn et al. reported an adult case with biallelic variations in *ZAP70* and *RNF168* having had a pediatric presentation (15). Another case report described developmental delay, and hearing loss in a patient with 3M syndrome due to co-existence variants in CUL7 and ILDR1 gene (16). Also, Amato et al. reported a case of Angelman syndrome (5,5 Mb deletion of 15q11.2–q13.1) with a coexisting intermediate junctional epidermolysis bullosa (COL17A1, c.3766 + 1G > A, homozygous) and autosomal recessive deafness type 57 (PDZD7, c.883C > T, homozygous) (17). This “double trouble” case illustrates the point that clinical findings in patients might not be explained by a single genetic defect, and consanguineous marriage increases the likelihood of coexistence of autosomal recessive diseases. Clinicians should consider exome or whole genome sequencing to identify disease-causing genetic defects in patients with potentially heterogenous clinical findings.

## Data Availability

The authors acknowledge that the data presented in this study must be deposited and made publicly available in an acceptable repository, prior to publication. Frontiers cannot accept a manuscript that does not adhere to our open data policies.
