# Supplementary material for: Case report: Artemis deficiency and 3M syndrome—coexistence of two distinct genetic disorders
Source: Front Pediatr. 2023 Jul 13;11:1211254. doi: 10.3389/fped.2023.1211254 (PMC10373501; doi:10.3389/fped.2023.1211254)
Supplement: Supplementary file 1 [file Image1.pdf]

A.

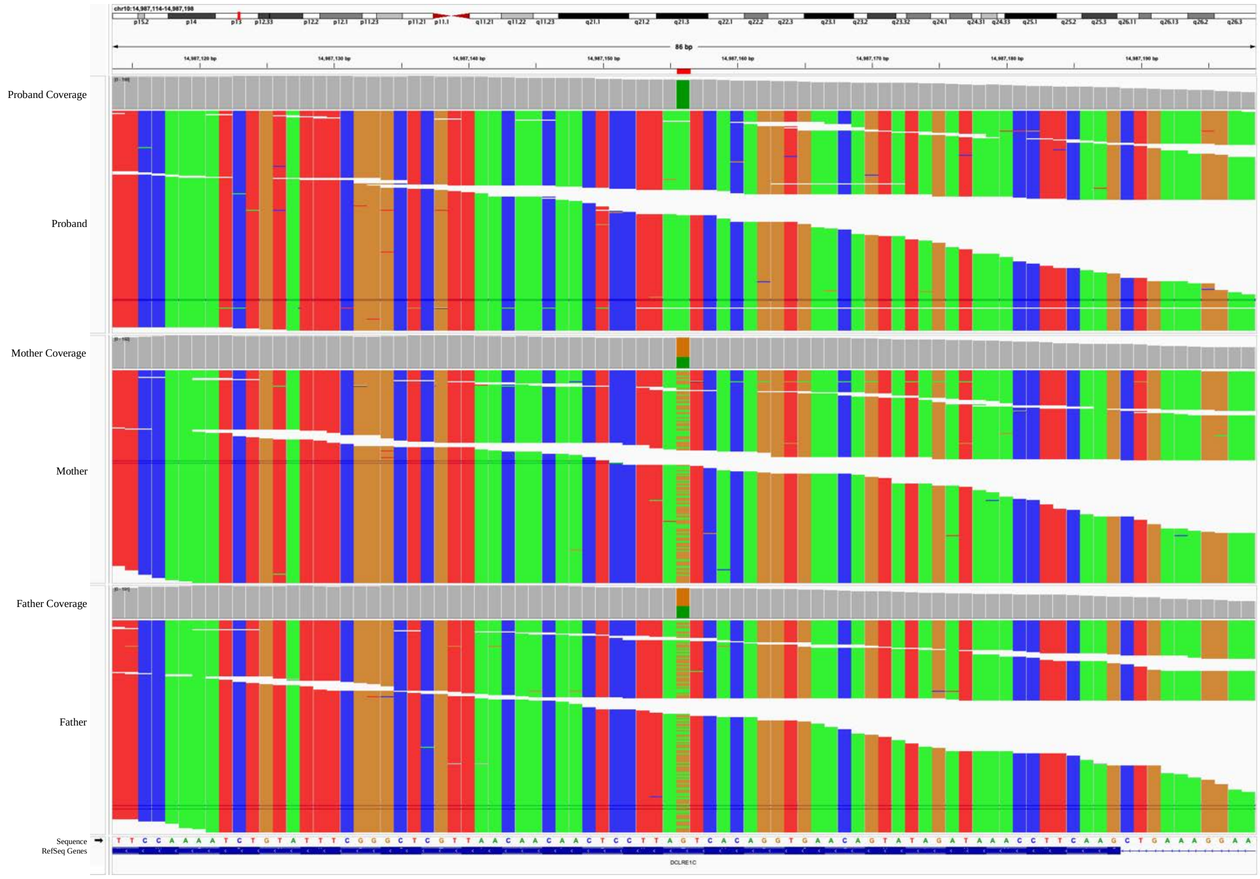
chr10:14,987,114-14,987,198
86 bp
Proband Coverage
Proband
Mother Coverage
Mother
Father Coverage
Father
Sequence
RefSeq Genes
DCLRE1C

B.

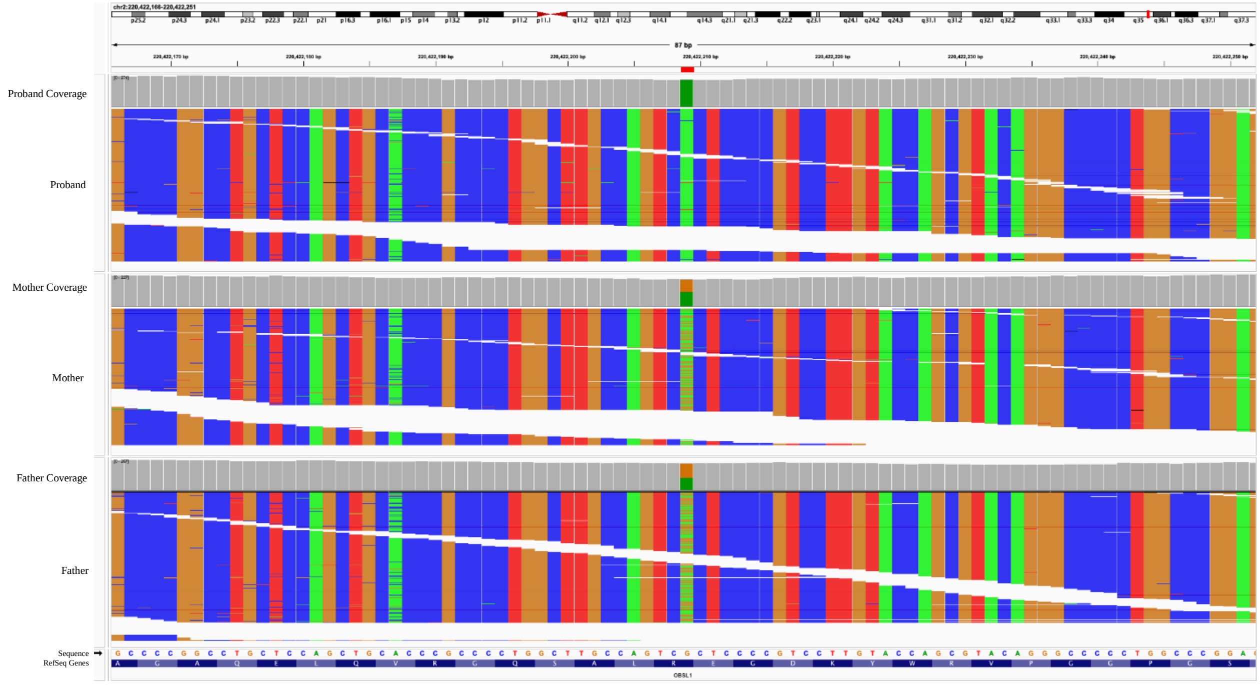
chr2:220,422,166-220,422,251
87 bp
Proband Coverage
Proband
Mother Coverage
Mother
Father Coverage
Father
Sequence
RefSeq Genes
OBSL1

Supplemental Figure: Binary Alignment Mapping (BAM) data for
A. *DCLRE1C*:c.194C>T:p.T65I and **B.** *OBSL1*:c.3922C>T:p.R1308X. Data are visualized using Integrative Genomics Viewer (IGV) version 2.16.1. Both plots show appropriate read-depth and quality at the loci of interest and confirm heterozygosity of each parent along with homozygosity of the proband.
